# The Influence of Age, Sex, and Socioeconomic Status on Glycemic Control Among People With Type 1 and Type 2 Diabetes in Canada: Patient-Led Longitudinal Retrospective Cross-sectional Study With Multiple Time Points of Measurement

**DOI:** 10.2196/35682

**Published:** 2023-04-27

**Authors:** Seyedmostafa Mousavi, Dana Tannenbaum Greenberg, Ruth Ndjaboué, Michelle Greiver, Olivia Drescher, Selma Chipenda Dansokho, Denis Boutin, Jean-Marc Chouinard, Sylvie Dostie, Robert Fenton, Marley Greenberg, Jonathan McGavock, Adhiyat Najam, Monia Rekik, Tom Weisz, Donald J Willison, Audrey Durand, Holly O Witteman, Diabetes Action Canada Research Questions Prioritization Study

**Affiliations:** 1 Diabetes Action Canada Toronto, ON Canada; 2 VITAM Research Centre in Sustainable Health Québec, QC Canada; 3 Université Laval Québec, QC Canada; 4 Centre de recherche sur le Vieillissement Sherbrooke, QC Canada; 5 School of Social Work Faculty of Letters and Human Sciences Université de Sherbrooke Sherbrooke, QC Canada; 6 Department of Family and Community Medicine North York General Hospital Toronto, ON Canada; 7 Department of Family and Community Medicine Temerty Faculty of Medicine University of Toronto Toronto, ON Canada; 8 Office of Education and Professional Development Faculty of Medicine Université Laval Québec, QC Canada; 9 Centre de recherche du CHUS Sherbrooke, QC Canada; 10 National Indigenous Diabetes Association Winnipeg, MB Canada; 11 Pediatrics and Child Health Rady Faculty of Health Sciences University of Manitoba Winnipeg, MB Canada; 12 Department of Operations and Decision Systems Faculty of Business Administration Université Laval Québec, QC Canada; 13 Cardiometabolic Health Diabetes and Obesity Research Network (CMDO) Sherbrooke, QC Canada; 14 Interuniversity Research Centre of Enterprise Networks (CIRRELT) Montréal, QC Canada; 15 Wounds Canada North York, ON Canada; 16 Diabetes Canada Toronto, ON Canada; 17 Institute of Health Policy Management and Evaluation University of Toronto Toronto, ON Canada; 18 Canada CIFAR AI Chair Québec, QC Canada; 19 Institute Intelligence and Data Université Laval Québec, QC Canada; 20 Department of Computer Science and Software Engineering Faculty of Science and Engineering Université Laval Québec, QC Canada; 21 Department of Electrical Engineering and Computer Engineering Faculty of Science and Engineering Faculty of Science and Engineering Québec, QC Canada; 22 Department of Family and Emergency Medicine Faculty of Medicine Université Laval Québec, QC Canada; 23 See Acknowledgments

**Keywords:** adolescent, adult, cohort studies, co-design, diabetes mellitus, diabetes mellitus type 1, diabetes mellitus type 2, glycated hemoglobin, menopause, participatory medicine, patient engagement, postmenopause, premenopause, public and patient involvement, sex factors, socioeconomic disparities in health, user design, patient engagement, public and patient involvement

## Abstract

**Background:**

Clinical guidelines for most adults with diabetes recommend maintaining hemoglobin A_1c_ (HbA_1c_) levels ≤7% (≤53 mmol/mol) to avoid microvascular and macrovascular complications. People with diabetes of different ages, sexes, and socioeconomic statuses may differ in their ease of attaining this goal.

**Objective:**

As a team of people with diabetes, researchers, and health professionals, we aimed to explore patterns in HbA_1c_ results among people with type 1 or type 2 diabetes in Canada. Our research question was identified by people living with diabetes.

**Methods:**

In this patient-led retrospective cross-sectional study with multiple time points of measurement, we used generalized estimating equations to analyze the associations of age, sex, and socioeconomic status with 947,543 HbA_1c_ results collected from 2010 to 2019 among 90,770 people living with type 1 or type 2 diabetes in Canada and housed in the Canadian National Diabetes Repository. People living with diabetes reviewed and interpreted the results.

**Results:**

HbA_1c_ results ≤7.0% represented 30.5% (male people living with type 1 diabetes), 21% (female people living with type 1 diabetes), 55% (male people living with type 2 diabetes) and 59% (female people living with type 2 diabetes) of results in each subcategory. We observed higher HbA_1c_ values during adolescence, and for people living with type 2 diabetes, among people living in lower income areas. Among those with type 1 diabetes, female people tended to have lower HbA_1c_ levels than male people during childbearing years but higher HbA_1c_ levels than male people during menopausal years. Team members living with diabetes confirmed that the patterns we observed reflected their own life courses and suggested that these results be communicated to health professionals and other stakeholders to improve the treatment for people living with diabetes.

**Conclusions:**

A substantial proportion of people with diabetes in Canada may need additional support to reach or maintain the guideline-recommended glycemic control goals. Blood sugar management goals may be particularly challenging for people going through adolescence or menopause or those living with fewer financial resources. Health professionals should be aware of the challenging nature of glycemic management, and policy makers in Canada should provide more support for people with diabetes to live healthy lives.

## Introduction

### Background

Diabetes is a chronic condition with 2 common types: type 1 and type 2 [1]. Both types of diabetes are marked by elevated blood glucose levels, but their causes of onset and treatments are generally different [2], and type 1 is less common than type 2 [3]. A standard laboratory test for people with all types of diabetes is the hemoglobin A_1c_ (HbA_1c_) test. Unlike a single blood glucose measure, HbA_1c_ is a measure of approximate mean blood glucose over 2-3 months [4] that can be measured at any time of day [5] and is often used as a marker of overall glycemic control. Higher HbA_1c_ levels are associated with an increased prevalence of complications of diabetes, affecting the eyes, kidneys, heart, and nerves [1]. Clinical guidelines in Canada recommend maintaining HbA_1c_ levels ≤7% (≤53 mmol/mol) for most adults with diabetes and ≤7.5% (≤59 mmol/mol) for most children with diabetes [6].

Among people with diabetes, differences in HbA_1c_ levels are associated with sociodemographic characteristics, including age [7-10], socioeconomic status [11,12], and sex [9,13-23]. Specifically, HbA_1c_ levels tend to be higher among adolescents compared with other age groups [7-10] and lower among people with higher socioeconomic status (measured using postal codes) compared with those with lower socioeconomic status [11,12]. Studies comparing HbA_1c_ levels by sex have shown mixed results across countries, sometimes showing higher HbA_1c_ levels among those who are male [9], sometimes showing higher HbA_1c_ levels among those who are female [13-20], and sometimes showing no difference [21-23].

The relationships between HbA_1c_ levels and individual and social characteristics are not only apparent in the literature [24]; they are also tangible in the lives of people with diabetes. As noted by team members living with diabetes reflecting on their own lives and on comparisons with peers, HbA_1c_ goals may be easier to attain in some situations than others. Such expertise and perspective gleaned from the lived experience of diabetes can add insight and nuances to diabetes research. Including this expertise in health research is a central tenet of patient partnership. Patient-partnered research involves people living with conditions as full members of the research team. In addition to ethical reasons that people living with conditions should be included in research decisions that affect them, such inclusion can also improve the relevance and quality of the studies [25-27].

### Objective

This study’s research question was developed by people living with type 1 or type 2 diabetes who were involved in a larger project in which people living with diabetes developed and prioritized research questions about diabetes. This study aimed to answer one of the identified and prioritized questions*: Accounting for socioeconomic status, what patterns exist in HbA_1c_ results among people in Canada with type 1 or type 2 diabetes of different sexes at different ages?* Although some data already exist regarding HbA_1c_ results in Canada [24,28], there has not yet been a national analysis like this stratified by the type of diabetes and including variables of sex, age, and socioeconomic status. Furthermore, few or no studies have included people living with diabetes as members of the research team.

## Methods

### Study Design

This study used a longitudinal retrospective cross-sectional study design with multiple time points of measurement.

### Study Data and Inclusion Criteria

To answer our research question, we used data from the Canadian National Diabetes Repository. As of July 1, 2021, this repository consisted of electronic medical records of 123,543 people with type 1 and type 2 diabetes living in Canada. The repository continues to grow, and as of April 23, 2022, it included records of 147,459 such people. Records describe patients treated in family medicine practices in 5 Canadian provinces: Ontario (57% of HbA_1c_ results), Alberta (25% of HbA_1c_ results), Manitoba (15% of HbA_1c_ results), Quebec (3% of HbA_1c_ results), and Newfoundland and Labrador (0.4% of HbA_1c_ results) [29,30]. These data originate from a larger data repository, the Canadian Primary Care Sentinel Surveillance Network. Previous analyses of the representativeness of the Canadian Primary Care Sentinel Surveillance Network have noted that, compared with the Canadian population, the data describe more people who are older and more people who are female, which may reflect patterns of greater health care seeking among these groups. It is therefore recommended to include age and sex in analytical models when analyzing these data [31].

In the National Diabetes Repository, people are identified as having diabetes if their record includes an HbA_1c_ result of ≥6.5% or at least 2 fasting blood glucose results >7 mmol/L recorded on different dates ≤1 year apart. However, medical records in Canada do not currently specify the type of diabetes. Therefore, we distinguished between people with type 1 and type 2 diabetes using an algorithm recently developed and validated using Canadian data. The machine learning algorithm analyzed 21 variables (eg, insulin use, nonmetformin antihyperglycemic agent use, and insulin pump use) and demonstrated a sensitivity (ie, ability to correctly identify that someone with type 1 diabetes has type 1 diabetes) of 80.6% and a specificity (ie, ability to correctly identify that someone without type 1 diabetes does not have type 1 diabetes) of 99.8% [32].

For this study, we included all HbA_1c_ results from the Canadian National Diabetes Repository from individuals with diabetes who had at least one HbA_1c_ measurement between 2010 and 2019 and for whom our other variables of interest (age, sex, and socioeconomic status) were available. In other words, we excluded records that lacked one or more of these data elements. We also excluded records with HbA_1c_ results <3.5% (15 mmol/mol) or >20% (195 mmol/mol), as clinical experts on our team deemed these to be likely laboratory errors, data entry errors, or rare outliers. We derived individuals’ ages by subtracting each person’s date of birth from the date when each HbA_1c_ measurement was performed. We excluded data from individuals aged <10 years, as they contributed such a small proportion of the data in the repository (0.04%) that we would be unable to conduct robust analyses for this subpopulation. As a proxy measure of socioeconomic status, the Canadian National Diabetes Repository applies an established index developed by the Canadian Institute for Health Information [33] to derive neighborhood before-tax income quintiles using the first 3 digits of individuals’ most recent residential postal codes. Quintile 1 denotes people living in lower-income neighborhoods, quintiles 2 to 4 denote middle-income neighborhoods, and quintile 5 is assigned to the highest-income neighborhoods [34].

### Statistical Analysis

Our unit of analysis was each HbA_1c_ result. We graphically inspected HbA_1c_ results across age groups using locally estimated scatterplot smoothing curves for type 1 diabetes. We used generalized additive model curves for type 2 diabetes to account for the large amount of data in this category. We then analyzed HbA_1c_ results using generalized estimating equations to account for dependency structure at the between-subject and within-subject levels. As the correlations between HbA_1c_ measurements in different years were similar, we used an exchangeable correlation structure to account for correlations between HbA_1c_ measurements of each individual [35]. As the distribution of HbA_1c_ levels was highly skewed, we used log transformation to reduce the skewness and stabilize the variance [36]. Owing to nonlinearity, we categorized age, dividing it into ordinal categories at 10- or 20-year intervals. The age range of 10 to 19 years included puberty and menarche for a large proportion of people who experience puberty and menarche [37,38]. The age range of 20 to 39 years included childbearing years for a large proportion of people who experience pregnancy [39-41]. The age range of 40 to 59 years included perimenopause and menopause for a large proportion of people who experience perimenopause and menopause [42-45]. The age range of 60 to 79 years included postmenopause for a large proportion of people who experience postmenopause [46]. The age range of ≥80 years included advanced adulthood.

We performed 2-way ANOVA to determine the relationship between HbA_1c_ levels and independent variables. We then analyzed potential interactions between age and sex for type 1 and type 2 diabetes, reporting pairwise interaction contrasts. We set our threshold for statistical significance at *P*<.05. We conducted 2 sets of sensitivity analyses. First, to explore whether practice and guideline changes over time might have influenced HbA_1c_ results and our findings, we conducted sensitivity analyses by rerunning all plots and models on 3 subsets of data: 2010 to 2012, 2013 to 2016, and 2017 to 2019. Second, we wished to check for potential biases in our results associated with unstable blood glucose levels shortly after diabetes diagnosis. In other words, if diagnostic HbA_1c_ values were present in the data set, higher HbA_1c_ values may not have anything to do with diabetes care or self-management but rather, may simply be reflections of a new condition. We did not have dates of diagnoses in the data set; therefore, to explore this issue, we removed the earliest HbA_1c_ result from the data set for each person and reran our analytical models. The details of these sensitivity analyses are provided in Multimedia Appendix 1.

We performed all analyses using R 3.6.1 (R Foundation for Statistical Computing) and associated packages, including ggplot2 for graphics, GEE for generalized estimating equations, and estimated marginal means for contrasts [47-57].

### Interpreting Results With Team Members With Diabetes

Following our analyses, we organized meetings with members of the research team living with type 1 and type 2 diabetes. These team members had already been involved throughout the yearlong larger project, including a series of early meetings to orient all team members to epidemiological cohort studies and subcommittee meetings led and attended only by patient partners to generate research questions.

To present and discuss the results of this study, our bilingual team held 1 meeting in English and 1 meeting in French. We invited all team members living with diabetes to attend, presented the results for approximately 15 minutes, and then held an open discussion for the remaining 45 minutes. We recorded the meetings to ensure that we accurately noted team members’ comments, and we invited all team members to review the manuscript to ensure that we conveyed meaning correctly. We invited all team members to fulfill the authorship criteria as defined by the International Committee of Medical Journal Editors and, accordingly, be coauthors of the manuscript. A total of 9 patient partner team members accepted the invitation to serve as coauthors of the manuscript.

Because team members were identified as such; that is, they were members of the research team, not study participants, neither our research team nor the research ethics committee that approved the study wished to treat these team members any differently than the team members who were professors, scientists, health professionals, students, and research staff. For this reason, we did not treat discussions among patient partners as research data with study participants, but rather, used the same approaches that one might use with any group of research team members. Specifically, we recorded meetings to ensure accurate note-taking, wrote summaries of discussions, and invited meeting attendees to review and comment on the summaries as we collaboratively drafted this manuscript. Although scientific papers do not typically report the identity characteristics of authors and it is rare to read concerns about whether a group of scientist authors can adequately represent the perspectives of the scientific community as a whole, we will also note that Diabetes Action Canada’s methods for recruitment of patient partners explicitly focuses on making extra efforts to ensure broad representation [27]. For this project, as in other projects [58], we deliberately constructed a team of people across all project roles who had a wide variety of backgrounds, with a particular focus on ensuring diversity in type of diabetes and in ethnocultural and socioeconomic backgrounds.

### Ethics Approval

Our study received ethics approval from the Laval University Research Ethics Committee, 2020-373/20-01-2021. The original data collection was also conducted with ethics approval. This study was approved by the National Diabetes Repository governance committee. The governance committee comprises a minimum of 50% of people living with diabetes.

## Results

### Population

The resulting data set consisted of 2 groups of data: one for people identified by the algorithm as having type 1 diabetes and one for people identified by the algorithm as having type 2 diabetes. Table 1 summarizes the HbA_1c_ results from people with type 1 and type 2 diabetes according to age, sex, and socioeconomic status. Because HbA_1c_ continues to be reported as a percentage in Canada, we use percentages to facilitate understanding by people living with diabetes in Canada. Of the 1950 and 946,931 available records, we excluded 1 record from the data available for people with type 1 diabetes and 1337 records from the data available for people with type 2 diabetes owing to HbA_1c_ values <3.5% (15 mmol/mol) or >20% (195 mmol/mol). Among people living with type 1 diabetes, individuals contributed data for a median of 3 (Q1-Q3 2-6) years. Most (244/296, 82.4%) participants contributed data to only 1 of the age groups used in our analyses. The others (52/296, 17.6%) contributed data to 2 age groups. Among people living with type 2 diabetes, individuals contributed data for a median of 5 (Q1-Q3 3-7) years. Most (68,437/90,417, 75.7%) participants contributed data to only 1 of the age groups used in our analyses. The others (21,980/90,417, 24.3%) contributed data to 2 age groups.

Mean HbA_1c_ was 8.3% (SD 1.7%) for female people with type 1 diabetes, 8.0% (SD 1.7%) for male people with type 1 diabetes, 7.1% (SD 1.3%) for female people with type 2 diabetes, and 7.2% (SD 1.3%) for male people with type 2 diabetes. In both types of diabetes, people who are female more often lived in geographic areas with lower socioeconomic status compared with people who are male.

**Table 1:** Characteristics of hemoglobin A_1c_ results^a^ among people with type 1 and type 2 diabetes.

Variable			Type 1 diabetes (1949 HbA_1c_^b^ results from 296 people)				Type 2 diabetes (945,262 HbA_1c_ results from 90,417 people)		
			Male (n=155)		Female (n=141)		Male (n=47,286)		Female (n=43,131)
**Population**									
	People with HbA_1c_ results in 2 age groups, n (%)	22 (14.2)		30 (21.3)		11,630 (24.6)		10,350 (24)	
**HbA_1c_ results^a^**									
	Mean (SD), %	8 (1.7)		8.3 (1.7)		7.2 (1.3)		7.1 (1.3)	
	Mean (SD), mmol/mol	64 (16)		67 (16)		55 (12)		54 (12)	
	Median (Q1-Q3^c^; range^d^), %	7.6 (6.9-8.5; 4.8-16.3)		8 (7.2-9.1; 5-16)		6.9 (6.3-7.8; 3.5-19.6)		6.8 (6.3-7.6; 3.5-19.5)	
	Median (Q1-Q3^c^; range^d^), mmol/mol	60 (52-69; 29-155)		64 (55-76; 31-151)		52 (45-62; 15-191)		51 (45-60; 15-190)	
	Results per person, median (Q1-Q3^c^; range^d^)	4 (2-9; 1-42)		5 (2-9; 1-48)		8 (4-15; 1-112)		8 (4-15; 1-115)	
	HbA_1c_ results ≤7%, %	30.5		21		55		59	
	Total, n (%)	970 (49.7)		979 (50.2)		505,364 (53.4)		439,898 (46.5)	
**Age (years), n (%)^a^**									
	10-19	44 (4.5)		37 (3.7)		1020 (0.2)		1097 (0.2)	
	20-39	454 (46.8)		559 (57)		13,744 (2.7)		18,675 (4.2)	
	40-59	305 (31.4)		277 (28.2)		145,393 (28.7)		123,621 (28.1)	
	60-79	167 (17.2)		106 (10.8)		287,045 (56.7)		235,044 (53.4)	
	≥80	0 (0)		0 (0)		58,162 (11.5)		61,461 (13.9)	
**Socioeconomic status, n (%)^a^**									
	1 (lowest income)	199 (20.5)		336 (34.3)		115,565 (22.8)		116,715 (26.5)	
	2	141 (14.5)		151 (15.4)		108,374 (21.4)		98,284 (22.3)	
	3	223 (22.9)		196 (20)		94,672 (18.7)		80,026 (18.1)	
	4	222 (22.8)		182 (18.5)		93,043 (18.4)		74,537 (16.9)	
	5 (highest income)	185 (19)		114 (11.6)		93,710 (18.5)		70,336 (15.9)	

^a^As noted in the *Methods* section, our unit of analysis is each HbA_1c_ result. These summary statistics are therefore calculated across HbA_1c_ results in the data set, meaning that each HbA_1c_ result within a given category contributes 1 data point.

^b^HbA_1c_: hemoglobin A_1c_.

^c^Q1-Q3: quartile 1-quartile 3.

^d^Range=minimum value-maximum value.

**Figure 1:**
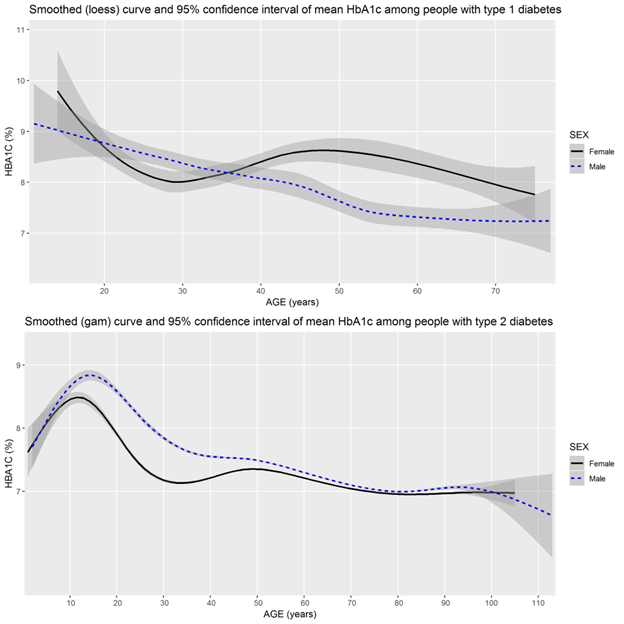
Mean HbA_1c_ levels by sex (male, female) across all ages of people with type 1 and type 2 diabetes in Canada. gam: generalized additive model; HbA_1c_: hemoglobin A_1c_; loess: locally estimated scatterplot smoothing.

**Figure 2:**
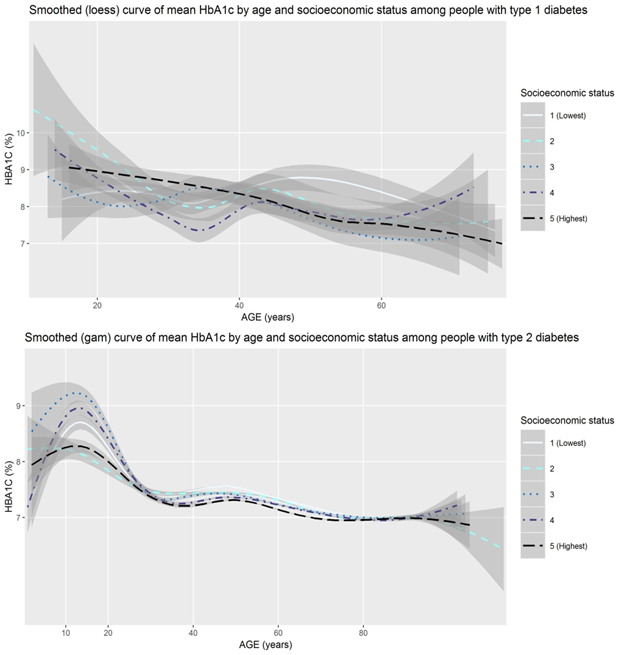
Mean HbA_1c_ levels of people with type 1 and type 2 diabetes in Canada across all ages by socioeconomic status. gam: generalized additive model; HbA_1c_: hemoglobin A_1c_; loess: locally estimated scatterplot smoothing.

As shown in Figure 1, we observed a relationship between age and HbA_1c_ levels among people with type 1 or type 2 diabetes. We also observed that these relationships may differ between people who are male and people who are female. As shown in Figure 2, socioeconomic status may also be associated with HbA_1c_ levels during much of adulthood, with somewhat higher HbA_1c_ values among adults aged 30 or 40 years through 70 years and living in geographic areas with lower mean income.

Table 2 shows the results of ANOVA fitted by generalized estimating equations for both types of diabetes.

According to our analyses of 296 people in the Canadian National Diabetes Repository with type 1 diabetes, there was a statistically significant relationship between age and HbA_1c_ levels, with overall lower HbA_1c_ levels among older people with type 1 diabetes. We observed no statistically significant relationships between sex and HbA_1c_, socioeconomic status and HbA_1c_, and the interaction term showed no statistically significant differences between people who are male and female at different ages. For 90,417 people in the Canadian National Diabetes Repository with type 2 diabetes, all variables demonstrated statistically significant relationships with HbA_1c_ levels. HbA_1c_ results are lower among people who are older, higher among people who are male, and higher among people living in geographic areas with lower mean income. There was a significant interaction between age and sex, suggesting that the pattern of lower HbA_1c_ values among older people with type 2 diabetes was somewhat different between those who are male and female. Specifically, those who are male appear to reach lower HbA_1c_ values later in their life span compared with those who are female. Multimedia Appendix 1 shows pairwise interaction contrasts for this interaction.

Sensitivity analyses (Multimedia Appendix 1) demonstrated that the results were similar for data from 2010 to 2012, 2013 to 2016, and 2017 to 2019, suggesting that practice and guideline changes during the data collection period did not substantially influence our findings. Sensitivity analyses also suggested no changes in our findings when the first HbA_1c_ value for each person was removed from the data set, suggesting that patterns observed are not a reflection of new diagnoses.

**Table 2:** Relationship between age, sex, socioeconomic status, and hemoglobin A_1c_ (HbA_1c_) among people with type 1 and type 2 diabetes in Canada.

	Type 1 diabetes (1949 HbA_1c_ results from 296 people)			Type 2 diabetes (945,262 HbA_1c_ results from 90,417 people)	
	*F* statistic	*P* value	*F* statistic		*P* value
Age	3.74	.01	635.70		<.001
Sex	2.26	.13	186.85		<.001
Socioeconomic status	0.77	.54	218.78		<.001
Age:sex interaction	1.48	.21	113.165		<.001

### Interpretation of Results by Team Members With Diabetes

Approximately equal numbers of team members living with type 1 and type 2 diabetes attended the meetings to discuss and interpret results. In these meetings, people living with diabetes raised potential post hoc explanations for the findings, asked technical questions about the analyses, raised potential study limitations, and discussed implications for policy and future research.

Specifically, with respect to explanations of findings, women living with type 1 diabetes suggested that the potential pattern among female people with type 1 diabetes mimicked their own life courses and may reflect lower HbA_1c_ levels during potential childbearing years and higher HbA_1c_ levels during potential menopausal years. People with diabetes also noted the differing sex-based differences between type 1 diabetes and type 2 diabetes. In both types of diabetes, HbA_1c_ results for people who are female dipped around the age of 30 years and peaked just before the age of 50 years, whereas HbA_1c_ values for people who are male decreased more smoothly with age. However, in the case of type 1 diabetes, curves met and intersected at multiple ages, whereas there were no similar meetings and intersections in the case of type 2 diabetes. Finally, people with diabetes questioned whether differences in the HbA_1c_ levels at different ages might reflect differences in the effort that people are able to put into diabetes management at different stages of their life course, depending on when they were diagnosed.

With respect to technical questions, people living with diabetes noted that accurate HbA_1c_ measurement was not possible for some individuals [59], queried how hypoglycemia unawareness might influence HbA_1c_ results, and raised the issue that HbA_1c_ is a highly imperfect measure. HbA_1c_ is essentially analogous to average blood glucose, and averages can mask substantial variation. Nonetheless, it remains a standard measure, as other methods of measurement (eg, time-in-range measured by flash or continuous glucose monitoring) are not universally available across Canada.

People living with diabetes also raised study limitations including the relatively small amount of data available for people with type 1 diabetes in these primary care electronic medical records, the need to use an algorithm to estimate diabetes type because of the lack of specificity about this in the Canadian electronic medical records, the lack of data on education level to better identify the contribution of income and education to glycemic control, and the lack of data on race and ethnicity, which strongly impact outcomes for people with diabetes in Canada and elsewhere. Team members with diabetes also questioned whether HbA_1c_ results might have differed during the 10-year span of the study given the introduction of new technologies in Canada between 2010 and 2019 that allow greater glycemic control.

With respect to implications for policy and future research, people living with diabetes noted that an HbA_1c_ level of ≤7% appeared to be very difficult to reach and maintain for many people living with diabetes in Canada. They suggested that these results be communicated to health professionals to help set realistic expectations and that people living with diabetes should consider being “insistent” with their health professionals to explore options for treatment and appropriate goals. Although guidelines suggest that HbA_1c_ targets should be set between a health professional and an individual living with diabetes while taking into account all relevant aspects of the individual’s life, in practice, many people with diabetes do not receive this level of individualized care [24].

Indigenous patient partners expressed interest in data specifically for Indigenous peoples in Canada. The National Diabetes Repository does not currently contain data from health centers specifically serving Indigenous communities, although there may be data from urban Indigenous people within the data set that cannot be identified separately from the larger data set. Our goal with this project was to establish a means for people living with diabetes to determine research questions and drive epidemiological cohort studies. Although we knew our data source would not allow us to answer research questions specific to Indigenous peoples, we specifically included Indigenous patient partners, researchers, and non-Indigenous researchers who work with Indigenous communities in the project to help us collectively ensure that our approaches would not harm potential future Indigenous-led projects conducted under relevant ethical frameworks such as the First Nations’ Principles of Ownership, Control, Access, and Possession [60].

## Discussion

### Principal Findings

In this study co-designed with people living with diabetes, we aimed to explore differences in HbA_1c_ levels between people with type 1 or type 2 diabetes of different sexes at different ages and with different levels of socioeconomic status, using a large database of primary care electronic medical records for people with diabetes in Canada. We report 6 main observations from our study.

First, the differences in statistical significance between the smaller sample of people with type 1 diabetes and the much larger sample of people with type 2 diabetes are reflective of broader patterns in research. These patterns have policy implications that can affect the lives of people living with more or less common conditions, including more or less common types of diabetes. Larger sample sizes allow identification of smaller associations or effects [61]. In the case of diabetes, this means that it is easier to identify statistically significant effects in the much larger populations of people with type 2 diabetes than in the smaller populations of people with type 1 diabetes or the even smaller populations of people with other types. This can have negative policy impacts on people with diabetes, for different reasons. For people with type 2 diabetes, as health research enters the era of big data and personalized medicine, large data sets analyzed by research teams with little clinical, epidemiological, or personal expertise may allow identification of associations or effects that may not be clinically or personally meaningful. For people with type 1 diabetes, who represent an estimated 5% to 10% of cases of diabetes, minority status within the larger disease community has led to policy issues such as type 2 diabetes being identified as a risk factor for severe COVID-19 outcomes, whereas type 1 diabetes, which demonstrated higher odds ratios or hazard ratios for severe COVID-19 outcomes in multiple studies but had wider CIs owing to smaller populations [62-64], was identified only as a “potential risk factor” [65]. As with other less common conditions, it is important that policy decisions that affect people with diabetes account for differences in type of diabetes and avoid applying the same statistical standards to differently sized populations without accounting for the influence of sample size.

Second, age is an important consideration for HbA_1c_ targets for people with both types of diabetes. Similar to studies in other countries [7-10,66,67], our study demonstrated overall higher HbA_1c_ values among adolescents with diabetes compared with people with diabetes in other age groups. This may reflect the ways in which adolescents differ from people in other age groups due to biology (eg, puberty, menarche), diabetes management (eg, time since diagnosis to develop useful habits and patterns, lack of full control over management options due to family preferences and finances), and life stage (eg, externally imposed structures of school, work, family; internally-directed focus on social development). Although providing high-quality health care to children and adolescents with diabetes has long been an area of focus in Canada [68], our results suggested that adolescents may still need more support both within and outside of clinical encounters.

Third, there is a tendency toward different patterns across the life course between people of different sexes with type 1 diabetes. Our relatively small sample of people with type 1 diabetes in this data set did not allow us to draw definitive conclusions. However, women living with type 1 diabetes in our team noted that the potential pattern we observed mimicked their own HbA_1c_ patterns during childbearing, perimenopausal, and menopausal years. Hermann et al [69] similarly observed significantly higher HbA_1c_ among female people with type 1 diabetes compared with male people with type 1 diabetes before the age of 30 years and after the age of 50 years. People with type 1 diabetes who plan to bear children may be particularly motivated to maintain a lower HbA_1c_ level during their childbearing years because of more stringent recommendations regarding glycemic control during pregnancy [70,71] and societal, medical, and self-directed expectations regarding how pregnant people, especially those at increased risk, should prioritize the health of their offspring [72-75].

Following childbearing, the hormonal shifts of perimenopause and menopause combined with common life stressors of middle age and gendered parenting roles may explain the somewhat higher HbA_1c_ values among many middle-aged female people. As noted by the lead patient partner in this project (DG), the evidence available about menopause and type 1 diabetes is scarce. There are a small number of studies addressing age of menopause among people with type 1 diabetes [76-79] and associated health risks [80]. However, there is little evidence about how to manage one’s diabetes and other health concerns post menopause [81]. This evidence gap negatively impacts the lives of people with type 1 diabetes who progress through menopause.

Fourth, the direction of the overall sex-based differences we observed among people with type 2 diabetes differed from some previous studies in other countries. In our study using data from people in Canada, male people with type 2 diabetes had overall higher HbA_1c_ values, indicating a potentially higher risk of diabetes-related complications compared with female people. Other studies using data from people in Portugal [13], Brazil and Venezuela [15], Korea [19], and Spain [82] reported the opposite, with overall higher HbA_1c_ values among those who are female compared with those who are male. Studies in the United States [22] and the Netherlands [23] reported no sex-based differences in HbA_1c_ values, and a study in Sweden reported higher HbA_1c_ values in male people compared with female people [9]. All of these other studies either focused on type 2 diabetes or did not distinguish between types of diabetes, meaning that the data were necessarily drawn from people with type 2 diabetes, who constitute 95% of people living with diabetes globally according to the World Health Organization [1]. The lack of agreement among different studies regarding sex-based differences might occur because overall differences may be a product of both biology and gender equality as seen via social roles. The World Economic Forum’s Global Gender Gap Report offers data in support of the suggestion that gender roles may explain the different results regarding sex-based differences across countries. As measured by this index, the countries in which male people have lower HbA_1c_ values than female people (Spain, Korea, Portugal, Brazil, Venezuela) have lower mean gender equality (mean 0.723, SD 0.043) than the countries in which there was no difference (the United States and the Netherlands: mean 0.763, SD 0.001), which in turn had lower mean gender equality than countries in which male people had higher HbA_1c_ values (Canada, Sweden: mean 0.798, SD 0.036) [83]. In other words, in countries with better overall gender equality, female people may have better diabetes-relevant health outcomes relative to male people, while in countries with worse gender equality, female people with type 2 diabetes may have worse diabetes-relevant health outcomes relative to male people.

Fifth, people with type 2 diabetes living in less affluent areas in Canada had higher HbA_1c_ levels than those living in more affluent areas. This is unfortunately unsurprising, as type 2 diabetes is a progressive disease and is more prevalent in Canada among people with lower incomes than among those with higher incomes [84-86]. There may also be a similar pattern among people with type 1 diabetes that is not detectable in our relatively small sample. People with type 1 diabetes with higher incomes have less variability in their HbA_1c_ results [12]. Lower income has been shown to be associated with higher HbA_1c_ among children with type 1 diabetes in Canada [87], and complications are more prevalent among people living with type 1 diabetes in Canada with lower incomes [88]. Owing to the uneven coverage of diabetes medications and devices across Canada, unequal access to high-quality health care, and large differences in levels of food security, people with both types of diabetes who live on lower incomes may face additional challenges in diabetes management compared with those with higher incomes [89-92]. Without efforts to address these inequities, such patterns may worsen in the coming years with the advent of new technologies and medications.

Finally, a substantial proportion of people with diabetes in Canada have not yet demonstrated guideline-recommended HbA_1c_ values. This shows the difficulty in reaching and maintaining this goal [93]. Similarly, Aronson and colleagues [94] showed, within a larger sample of 3600 adults living with type 1 diabetes and receiving care from endocrinologists in Canada, that less than a quarter of people demonstrated HbA_1c_ values ≤7%. Health professionals and policy makers should be aware of this gap to better support people living with diabetes in Canada. As noted by the people on our team who live with diabetes, health professionals’ acknowledgment of the difficulty of attaining this target would help them feel less like they are failing and more like they are part of a large group living with a difficult condition and potentially struggling to achieve targets set by academic researchers and health professionals. Policy makers can improve this situation by better funding health care, education, medications (eg, insulin and other medications), supplies (eg, test strips, flash or continuous glucose monitors, and closed-loop artificial pancreas systems) [95], food security initiatives (eg, access to affordable healthy foods) [96], healthy environment initiatives (eg, walking trails, bicycle paths, and community gardens) [97], broader antipoverty initiatives [98], and research aimed at supporting people living with diabetes in Canada to achieve self-directed health goals [99].

This study had 3 main limitations. First, although the data set was large and of overall high quality, the lack of relevant data (eg, electronic medical records in Canada do not include relevant data such as ethnicity), the use of proxy variables (eg, socioeconomic status as an unvarying quintile derived from the most recent postal codes), and the necessity of using an algorithm to predict the type of diabetes may have limited our results. We cannot be certain that all people predicted by the algorithm to have type 1 diabetes were correctly identified, and even then, the small number of people identified by the algorithm as having type 1 diabetes meant that our findings with respect to type 1 diabetes were less conclusive than those for type 2 diabetes. Other rarer types of diabetes such as latent autoimmune diabetes in adults were not represented at all. Second, we did not include comorbid medical conditions in this preliminary study, as these were not part of the research question identified by people living with diabetes. Although people who live with other conditions in addition to diabetes may have higher or lower HbA_1c_ values than those who live only with diabetes; for this preliminary analysis, we sought only to determine broad patterns in this large national data set. Third, our analyses had some threats to external validity (ie, generalizability). All medical records from the National Diabetes Repository were collected from the primary care records of 5 provinces in Canada. Approximately 15% of people in Canada lack access to a primary health care provider, and lack of access is not distributed evenly [100,101], meaning that our study may have had some selection bias.

This study had 3 main strengths. First, the entire study, from the initial idea and development of the research question to the interpretation of results and drafting of this manuscript, was conducted in full partnership with people living with the condition studied. This allowed us to identify a research question that is relevant to people living with diabetes in Canada, enrich our interpretation of results, and avoid framing our results in ways that are heedless of the humanity of people whose medical data were analyzed. Second, this study offered insight into glycemic control for people living with diabetes across Canada, included key variables of sex and age, and accounted for the potential influence of socioeconomic status. It is important to avoid a one-size-fits-all approach when discussing diabetes management. Identifying patterns according to common variables is a step toward more individualized care. Third, data from the Canadian National Diabetes Repository represent high-quality big data across multiple provinces, allowing national-level analyses that were previously difficult to perform in Canada.

### Conclusions

This study demonstrated the value and potential of patient-led research and of a national data repository for diabetes. People who live with a condition should have the power to set health research agendas so that research serves their needs. Responding to a research question developed by people living with diabetes, we mapped nearly a million HbA_1c_ results over 10 years from people with type 1 and type 2 diabetes in Canada and showed how HbA_1c_ results may differ by age, sex, and socioeconomic status. These factors are important to consider when setting HbA_1c_ targets and when studying the relationship between HbA_1c_ levels and complications of diabetes. Further research and support are needed to help people manage diabetes across life stages, with notable challenges during adolescence for people of both sexes and during menopause for people of female sex. As noted by members of our research team living with diabetes, people living with diabetes may not consistently receive adequate support from their health care team, family, employer, and regulatory and funding systems that determine the availability of medications and technologies. Health professionals should be aware of the difficulty in maintaining an HbA_1c_ value below the guideline-recommended targets without access to additional support, medications, and technologies. Policy makers should set policies that enable people with diabetes in Canada to live healthy lives.

## Appendix

Multimedia Appendix 1 Pairwise contrasts and sensitivity analyses.

## Abbreviations

HbA_1c_: hemoglobin A_1c_

## Data Availability

National Diabetes Repository data are available for analysis [102].
