# Supplementary material for: The Influence of Age, Sex, and Socioeconomic Status on Glycemic Control Among People With Type 1 and Type 2 Diabetes in Canada: Patient-Led Longitudinal Retrospective Cross-sectional Study With Multiple Time Points of Measurement
Source: JMIR Diabetes. 2023 Apr 27;8:e35682. doi: 10.2196/35682 (PMC10176138; doi:10.2196/35682)
Supplement: Multimedia Appendix 1 [file diabetes_v8i1e35682_app1.pdf]

Multimedia Appendix: Pairwise contrasts and sensitivity analyses

Pairwise Contrasts

Figure S1. Pairwise contrasts for sex differences in HbA1c results in each age categories

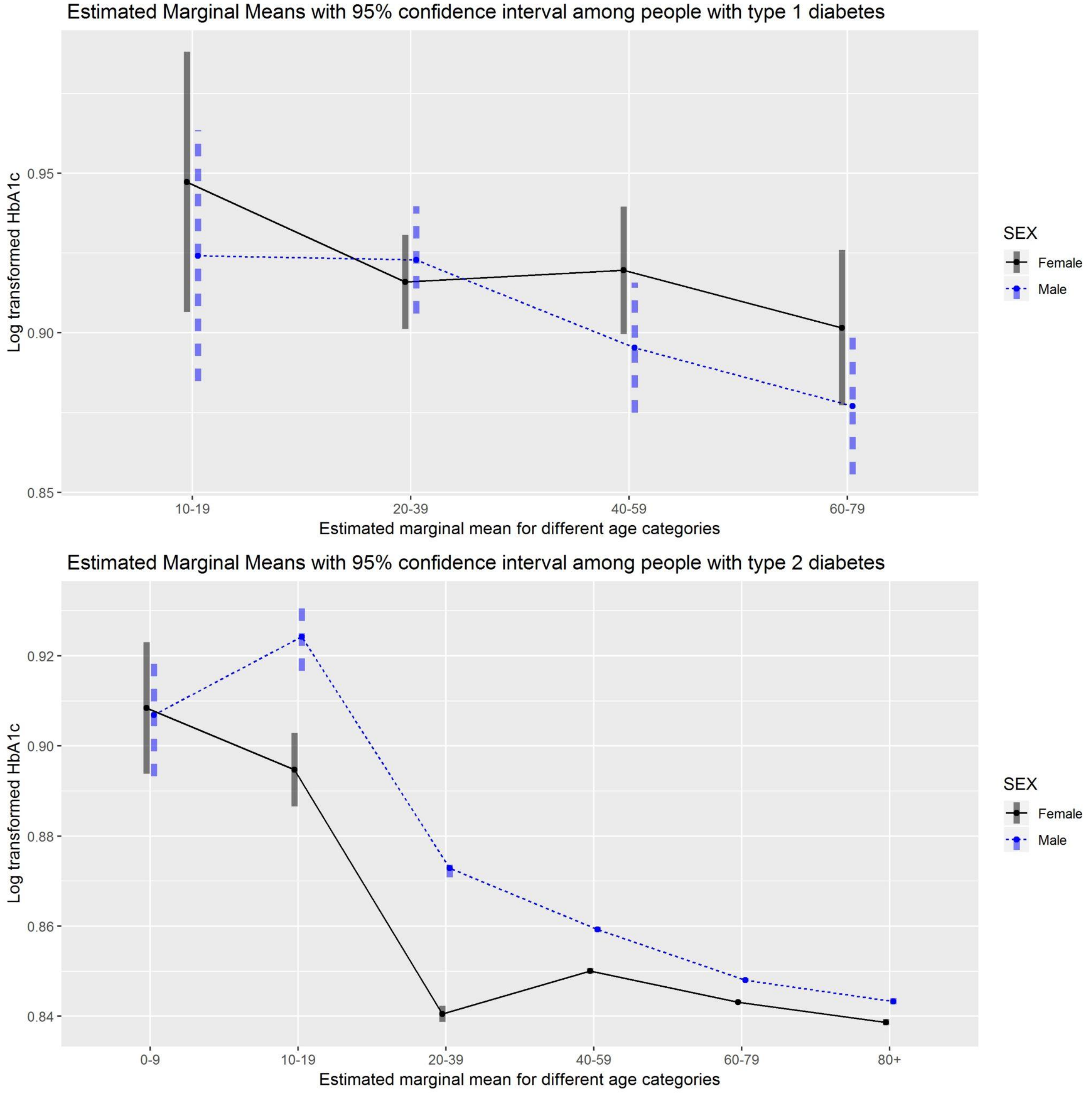

Table S1. Pairwise contrasts for sex differences in HbA1c results in people with type 1 and 2 diabetes in each age category

	Type 1 diabetes (total n = 1949 HbA1c results from 296 people)			Type 2 diabetes (total n = 945,262 HbA1c results from 90,417 people)		
Age Category	Estimate	Standard Error	P-value	Estimate	Standard Error	P-value
10 - 19	0.02314	0.0291	0.4260	-0.0295	0.00568	<.0001
20 - 39	-0.00689	0.0114	0.5450	-0.0324	0.00137	<.0001
40 - 59	0.02426	0.0146	0.097	-0.0092	0.00043	<.0001
60 - 79	0.02456	0.0165	0.1380	-0.0049	0.00027	<.0001
80+	-	-	-	-0.0046	0.0005	<.0001

Sensitivity Analysis: Changes in Practice and Guidelines

Figure S2. Mean HbA1c of male and female people across all ages of people with type 1 and type 2 diabetes in Canada: 2010-2012

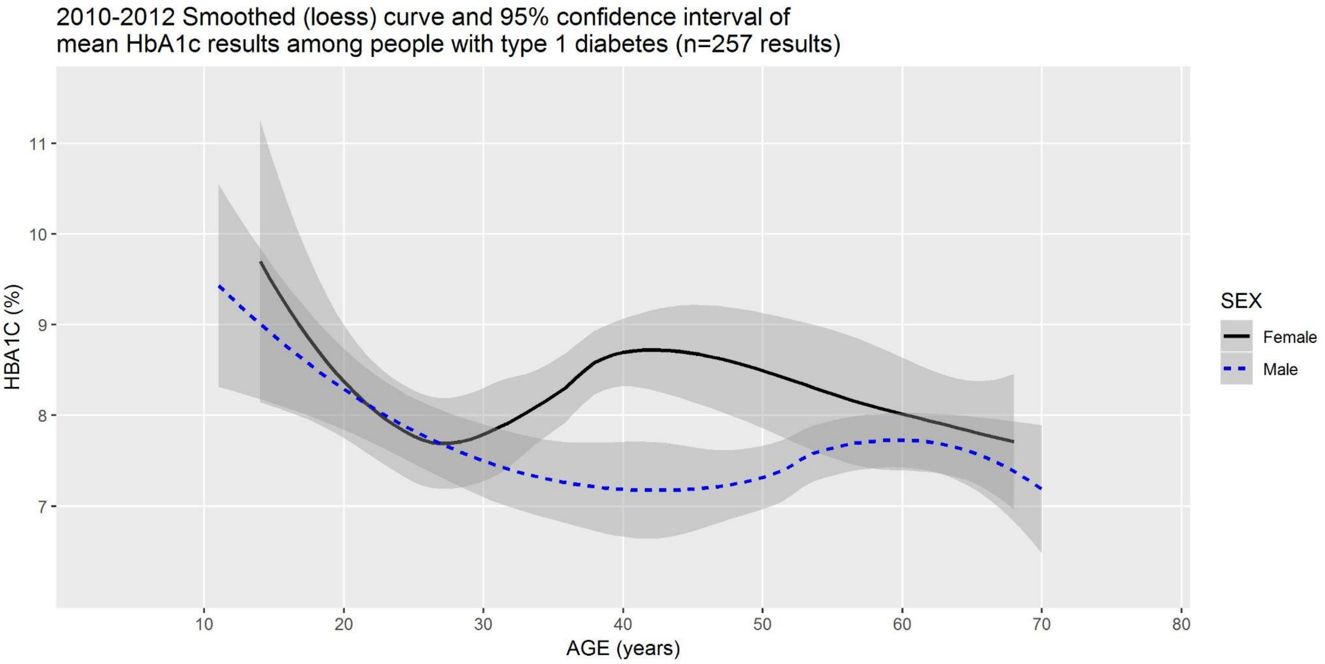

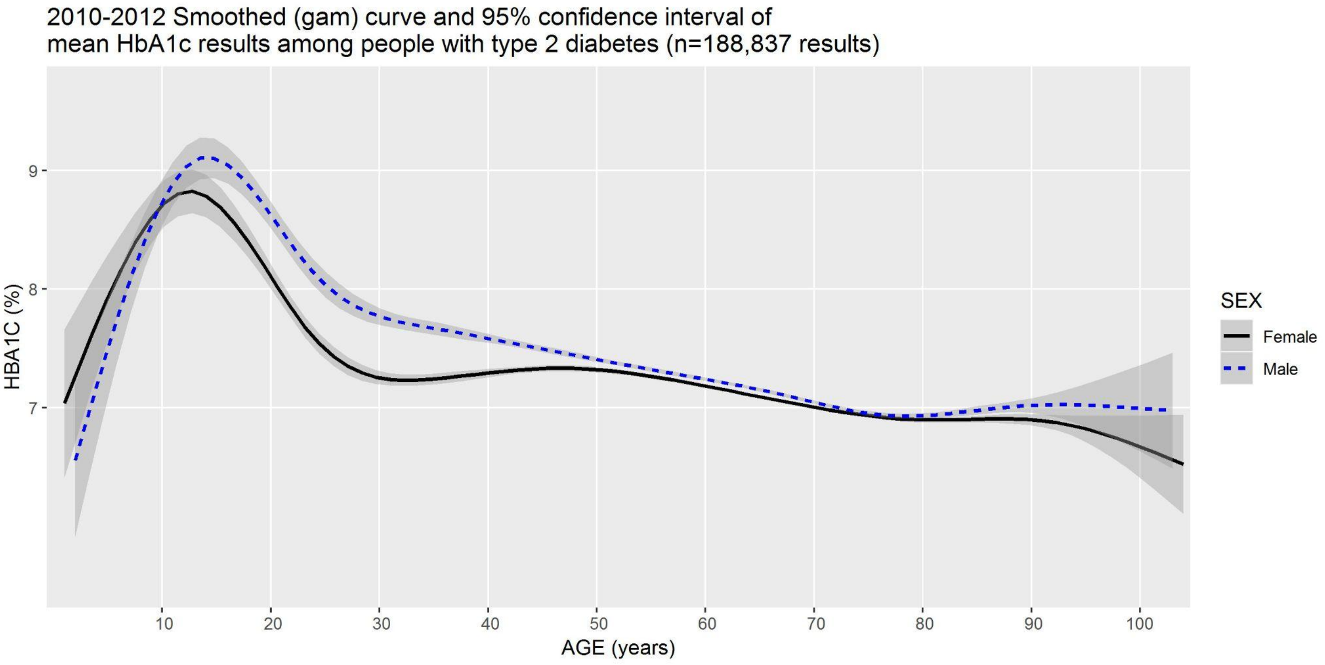

Figure S3. Mean HbA1c of male and female people across all ages of people with type 1 and type 2 diabetes in Canada: 2013-2016

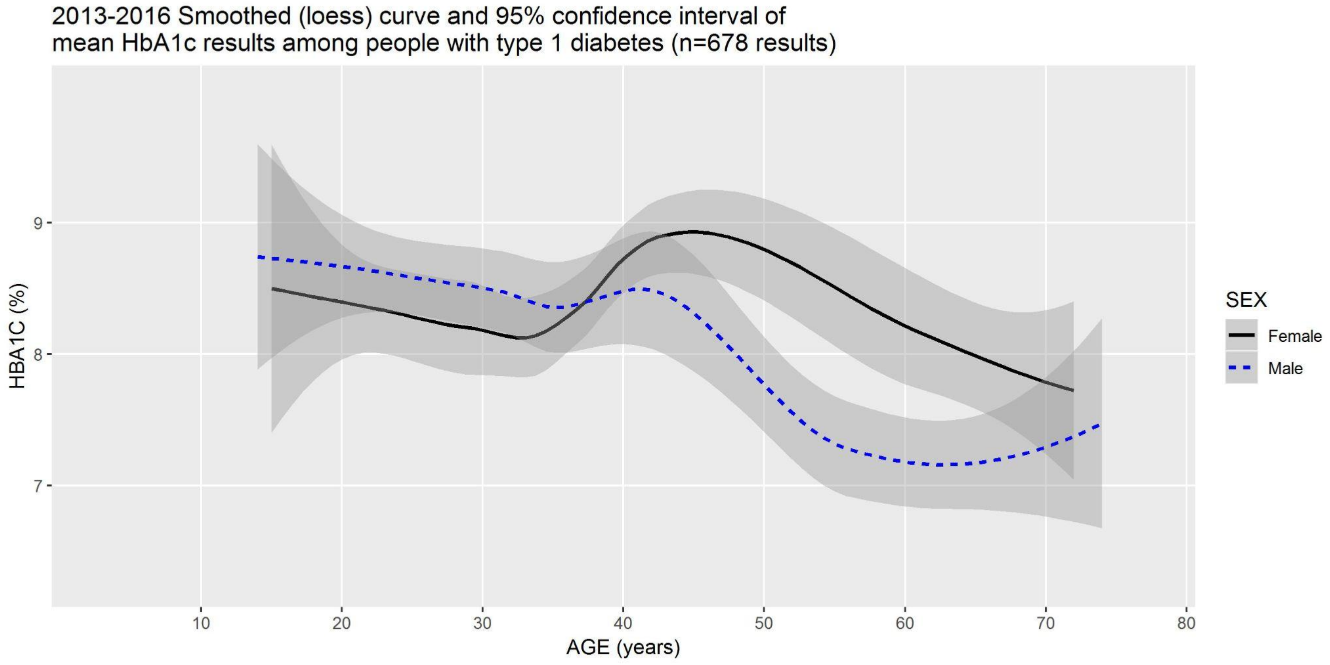

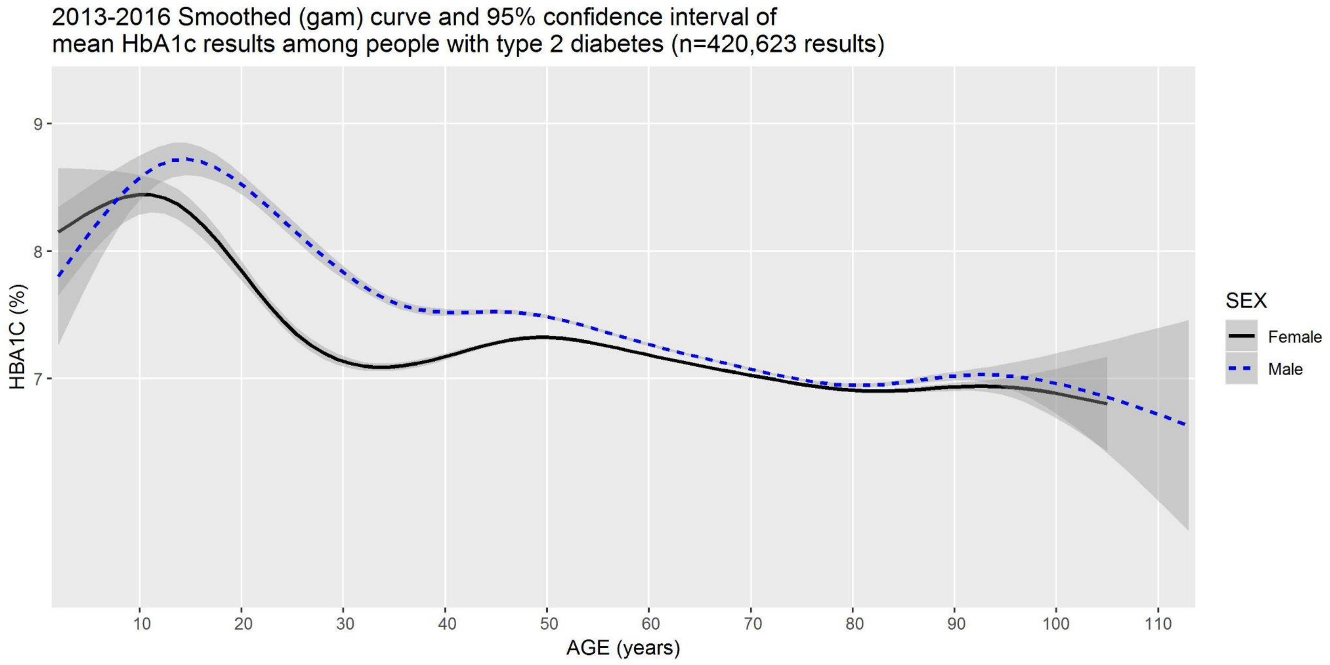

Figure S4. Mean HbA1c of male and female people across all ages of people with type 1 and type 2 diabetes in Canada: 2017-2019

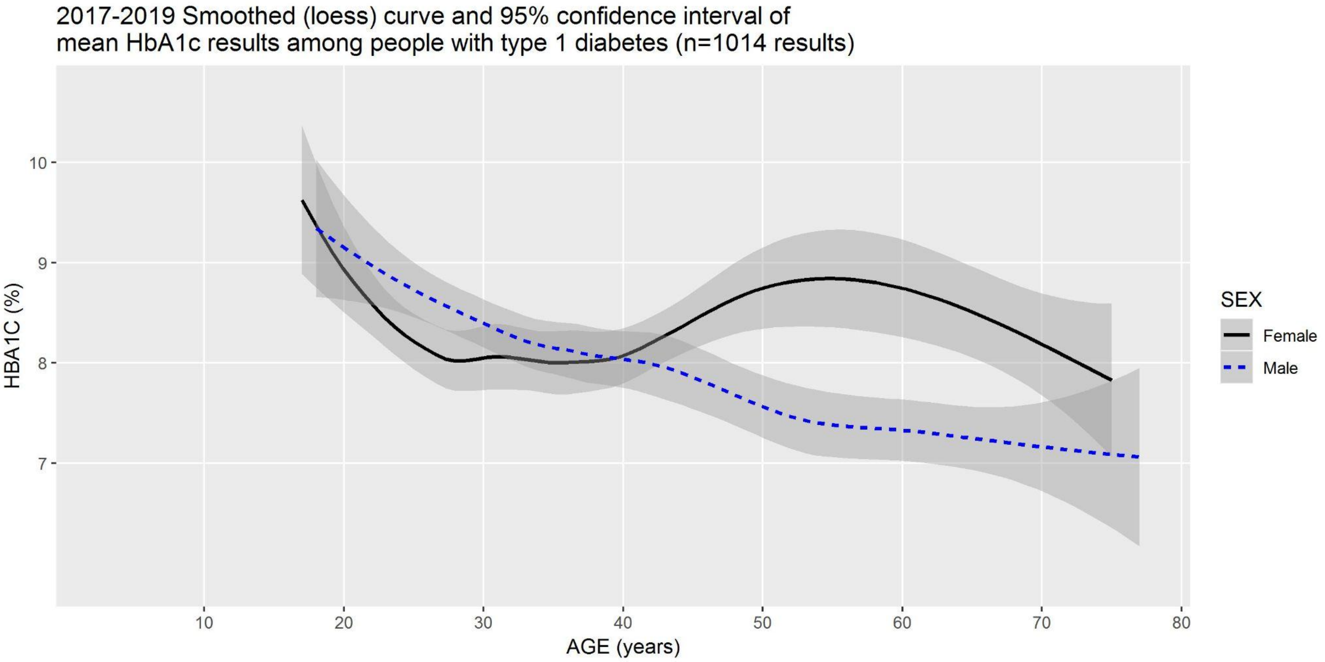

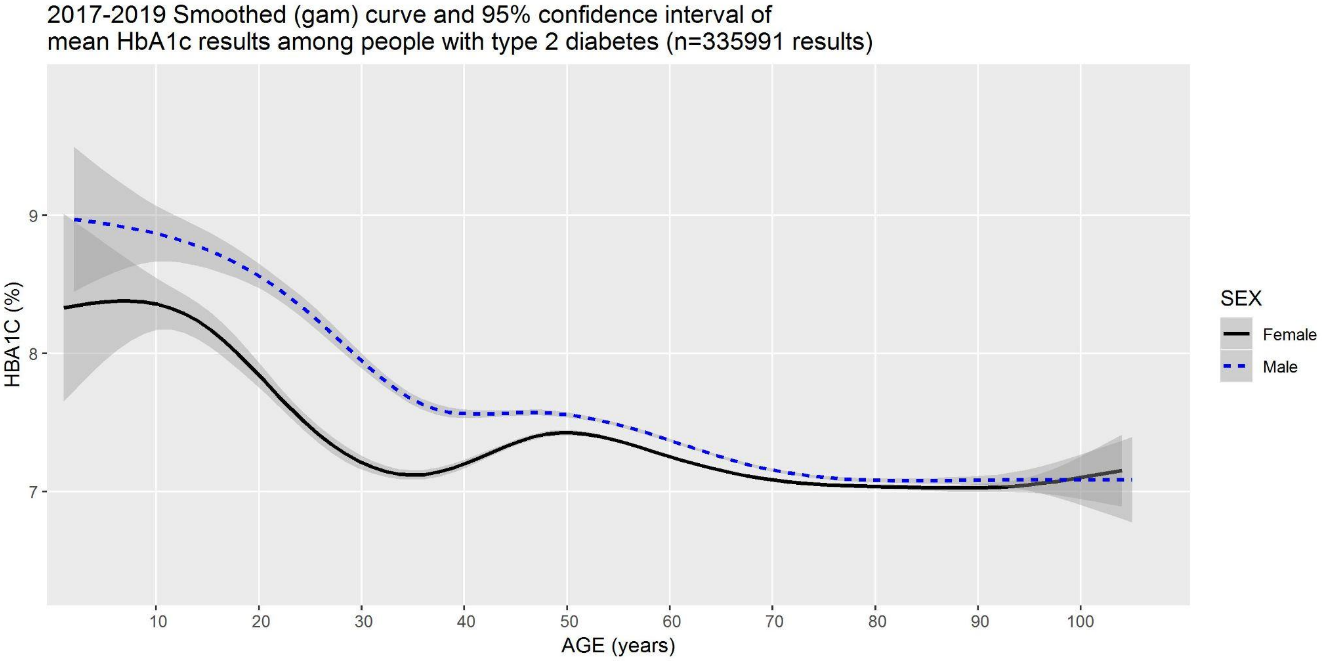

Table S2. Relationships between age, sex, socioeconomic status and HbA1c among people with type 1 and type 2 diabetes in Canada: 2010-2012

	Type 1 diabetes (total n = 257 HbA1c results from 77 people)		Type 2 diabetes (total n = 188,837 HbA1c results from 47,090 people)	
	F statistic	P value	F statistic	P value
Age	1.10	0.3475	257.452	<.0001
Sex	7.25	0.0071	37.377	<.0001
Socioeconomic Status	0.93	0.4435	40.189	<.0001
Age:Sex interaction	0.61	0.6043	22.887	<.0001

Table S3. Pairwise contrasts for sex differences in HbA1c results in people with type 1 and 2 diabetes in each age categories: 2010-2012

	Type 1 diabetes (total n = 257 HbA1c results from 77 people)			Type 2 diabetes (total n = 188,837 HbA1c results from 47,090 people)		
Age Category	Estimate	Standard Error	P-value	Estimate	Standard Error	P-value
10 - 19	0.0516	0.0359	0.1509	-0.02329	0.010546	0.0272
20 - 39	0.0233	0.0242	0.3364	-0.02999	0.002920	<.0001
40 - 59	0.0627	0.0261	0.0163	-0.00670	0.000867	<.0001
60 - 79	0.040	0.0217	0.0646	-0.00310	0.000548	<.0001
80+	-	-	-	-0.00452	0.001048	<.0001

Table S4. Relationships between age, sex, socioeconomic status and HbA1c among people with type 1 and type 2 diabetes in Canada: 2013-2016

	Type 1 diabetes (total n = 678 HbA1c results from 163 people)		Type 2 diabetes (total n = 420,623 HbA1c results from 73,296 people)	
	F statistic	P value	F statistic	P value
Age	2.542	0.0544	461.355	<.0001
Sex	0.433	0.5106	65.706	<.0001
Socioeconomic Status	0.705	0.5882	134.285	<.0001
Age:Sex	4.159	0.0059	56.584	<.0001

interaction				

Table S5. Pairwise contrasts for sex differences in HbA1c results in people with type 1 and 2 diabetes in each age categories: 2013-2016

	Type 1 diabetes (total n = 678 HbA1c results from 163 people)			Type 2 diabetes (total n = 420,623 HbA1c results from 73,296 people)		
Age Category	Estimate	Standard Error	P-value	Estimate	Standard Error	P-value
10 - 19	-0.02255	0.0336	0.5023	-0.02249	0.008527	0.0084
20 - 39	-0.00885	0.0153	0.5627	-0.03181	0.001962	<.0001
40 - 59	0.06349	0.0194	0.0011	-0.01017	0.000605	<.0001
60 - 79	0.00311	0.0225	0.8899	-0.00405	0.000374	<.0001
80+	-	-	-	-0.00322	0.000697	<.0001

Table S6. Relationships between age, sex, socioeconomic status and HbA1c among people with type 1 and type 2 diabetes in Canada: 2017-2019

	Type 1 diabetes (total n = 1014 HbA1c results from 274 people)		Type 2 diabetes (total n = 335,991 HbA1c results from 73,854 people)	
	F statistic	P value	F statistic	P value
Age	4.659	0.0029	535.204	<.0001
Sex	2.763	0.0965	56.442	<.0001
Socioeconomic Status	0.807	0.5203	84.852	<.0001
Age:Sex interaction	2.119	0.0955	46.747	<.0001

Table S7. Pairwise contrast for sex differences in HbA1c results in people with type 1 and 2 diabetes in each age categories: 2017-2019

	Type 1 diabetes (total n = 1014 HbA1c results from 274 people)			Type 2 diabetes (total n = 335,991 HbA1c results from 73,854 people)		
Age Category	Estimate	Standard Error	P-value	Estimate	Standard Error	P-value
10 - 19	0.00657	0.0601	0.2748	-0.5686	0.19747	0.004
20 - 39	-0.0115	0.0136	0.3967	-0.6180	0.04146	<.0001

40 - 59	0.0280	0.0159	0.0784	-0.1693	0.01275	<.0001
60 - 79	0.0295	0.0167	0.0767	-0.0919	0.00707	<.0001
80+	-	-	-	-0.0797	0.01294	<.0001

Sensitivity Analysis: Analyses Without Earliest HbA1c Results

Table S8. Relationships between age, sex, socioeconomic status and HbA1c among people with type 1 and type 2 diabetes in Canada with all first HbA1c results in the database removed

	Type 1 diabetes (total n = 1653 HbA1c results from 241 people)		Type 2 diabetes (total n = 854,845 HbA1c results from 83,107people)	
	F statistic	P value	F statistic	P value
Age	2.65	0.04	19.80	<.0001
Sex	1.81	0.17	54.45	<.0001
Socioeconomic Status	1.02	0.39	54.31	<.0001
Age:Sex interaction	0.68	0.55	12.20	<.0001

Table S9. Pairwise contrasts for sex differences in HbA1c results in people with type 1 and 2 diabetes in each age category with all first HbA1c results in the database removed

	Type 1 diabetes (total n = 1653 HbA1c results from 241 people)			Type 2 diabetes (total n = 854,845 HbA1c results from 83,107 people)		
Age Category	Estimate	Standard Error	P-value	Estimate	Standard Error	P-value
10 - 19	0.00592	0.0365	0.871	-0.02367	0.009231	<.0001
20 - 39	0.00534	0.0123	0.6651	-0.024	0.002253	<.0001
40 - 59	0.02746	0.0138	0.0466	-0.00897	0.000762	<.0001
60 - 79	0.02736	0.0162	0.0912	-0.00794	0.000544	<.0001
80+	-	-	-	-0.00816	0.000896	<.0001
